# Genomic Surveillance of BVDV in Southern Brazil: What Changed After a Decade in Rio Grande do Sul?

**DOI:** 10.3390/v18050498

**Published:** 2026-04-24

**Authors:** Leticia F. Baumbach, Raquel S. Alves, Laura J. Camargo, Eduardo O. Sanguinet, Leticia S. Santos, Lucas Marian, Gabriela E. Birlem, Roberto Schroeder, Fabiano Barreto, João Marcos N. Costa, Renata A. Casagrande, Matheus N. Weber, Cláudio W. Canal

**Affiliations:** 1 Laboratório de Virologia Veterinária, Faculdade de Veterinária, Universidade Federal do Rio Grande do Sul (UFRGS), Porto Alegre 90010-150, RS, Brazil; raquels.alves@hotmail.com (R.S.A.); laurajcamargo@gmail.com (L.J.C.); sanguineteduardo@gmail.com (E.O.S.); lt15ss2001@gmail.com (L.S.S.); claudio.canal@ufrgs.br (C.W.C.); 2 Laboratório de Patologia Animal, Centro de Ciências Agroveterinárias, Universidade do Estado de Santa Catarina (UDESC), Lages 88520-000, SC, Brazil; lucasmarian94@hotmail.com (L.M.); renata.casagrande@udesc.br (R.A.C.); 3 Laboratório de Imunologia e Biologia Molecular, Faculdade de Veterinária, Universidade Federal do Rio Grande do Sul (UFRGS), Porto Alegre 90010-150, RS, Brazil; gabibirlem@gmail.com (G.E.B.); matheus.weber@ufrgs.br (M.N.W.); 4 Laboratório Federal de Defesa Agropecuária (LFDA/RS), Ministério da Agricultura Pecuária e Abastecimento (MAPA), Porto Alegre 91780-580, RS, Brazil; roberto.schroeder@agro.gov.br (R.S.); fabiano.barreto@agro.gov.br (F.B.); 5 Secretaria de Defesa Agropecuária, Ministério da Agricultura Pecuária e Abastecimento (MAPA), Brasília 70043-900, MG, Brazil; joao.costa@agro.gov.br

**Keywords:** pestivirus, BVDV, molecular detection, phylogenetic analysis, genetic diversity, genomic surveillance

## Abstract

Bovine viral diarrhea virus (BVDV) is a major cattle pathogen associated with significant economic losses worldwide. In Brazil, the high genetic diversity of circulating strains represents an additional challenge for disease control. To update the molecular epidemiology of BVDV in southern Brazil, 16,198 bovine serum samples collected in 2020 through a national surveillance program were screened for pestivirus RNA by RT-qPCR. Forty-nine samples (0.30%) were positive and subjected to partial sequencing of the 5′UTR and N^pro^ regions. Phylogenetic analysis identified BVDV-1a (25/49; 51%), BVDV-1b (1/49; 2%), BVDV-1d (7/49; 14%), and BVDV-2b (16/49; 33%), with no detection of HoBiPeV. When compared descriptively with data from 2010 in the same region, BVDV-1a remained the most frequent subgenotype, while BVDV-2b also represented a substantial proportion of detections, contrasting with other regions worldwide. Although the two datasets are not directly comparable, and no statistically significant differences were observed, these findings provide an updated overview of circulating BVDV subgenotypes in Rio Grande do Sul. The absence of HoBiPeV contrasts with reports from other regions of Brazil and suggests a distinct regional pattern of pestivirus circulation. Overall, the results reinforce the importance of continuous genomic surveillance to monitor changes in viral diversity and support control strategies in cattle populations.

## 1. Introduction

Bovine viral diarrhea virus (BVDV) is one of the most economically significant pathogens of cattle, responsible for major losses in productivity, reproduction, and overall herd health [1]. Annual economic impacts exceed USD 1.5–2.5 billion in the United States alone [2], underscoring its relevance to global livestock production. Brazil, which is the home of the world’s largest cattle population and a leading beef exporter, remains particularly vulnerable to the impacts of BVDV [3]. BVDV infections range from subclinical to severe systemic disease, affecting the gastrointestinal, respiratory, and reproductive systems, and often causing immunodepression that predisposes animals to secondary infections [1]. When infection occurs during early gestation, before fetal immunocompetence, the virus can cross the placenta and establish persistent infection, leading to the birth of calves that remain viremic and continuously shed virus throughout life, serving as key reservoirs for viral maintenance and transmission within herds [4].

BVDV belongs to the genus *Pestivirus* (family *Flaviviridae*), with three main species affecting cattle: BVDV-1 (*Pestivirus bovis*), BVDV-2 (*Pestivirus tauri*), and HoBi-like pestivirus (HoBiPeV, *Pestivirus brazilense*) [5,6]. Within each species, multiple subgenotypes have been described with distinct geographic distributions. To date, at least 24 subgenotypes of BVDV-1 (from 1a to 1w) have been reported [7,8,9], while BVDV-2 is classified into four subgenotypes (2a–2d) [10]. The most recently recognized, BVDV-2d, was defined from a single contaminating isolate in fetal bovine serum from Argentina in 1995, and no additional members have been reported [11]. A further subgenotype, BVDV-2e, has recently been proposed [12]. HoBiPeV is suggested to comprise at least five subgenotypes (a–e) [13,14]. Recent taxonomic proposals suggest elevating the genus *Pestivirus* to the family level (*Pestiviridae*), reflecting its distinct evolutionary lineage within the order *Amarillovirales* [15].

The pestivirus genome is a positive-sense, single-stranded RNA molecule of approximately 12.3 kb, organized as a single open reading frame (ORF) flanked by untranslated regions (UTRs) at both the 5′ and 3′ ends. The ORF encodes a polyprotein processed into structural (C, Erns, E1, and E2) and nonstructural (N^pro^, p7, NS2-3, NS4A, NS4B, NS5A, and NS5B) proteins [16]. For molecular detection and phylogenetic analyses, the 5′UTR, N^pro^, and E2 regions are widely used due to their combination of conservation and sequence variability [17]. The 5′UTR is highly conserved and commonly employed for BVDV detection and represents the genomic region most frequently applied in phylogenetic analysis for initial classification [18]. The N^pro^ region, less conserved, allows subgenotype differentiation and reflects functional roles in immune modulation [19].

Several countries have implemented BVDV control or eradication programs, based on strict biosecurity, accurate diagnostics, and removal of persistently infected (PI) animals. BVDV is included in the list of notifiable diseases of the World Organization for Animal Health (WOAH) due to its potential for international spread [20]. These initiatives have enabled nations such as Austria, Denmark, Finland, Sweden, Norway, and most of Germany to achieve BVDV-free status. However, the absence of nationwide coordination in other regions limits progress [21,22]. In Brazil, where no national control program is currently in place, BVDV remains endemic, causing ongoing economic losses and animal health impacts. Implementing structured control measures, supported by genomic surveillance and periodic evaluation of vaccine strain compatibility, is essential to improve disease management and ensure effective immunization against locally circulating viral variants [23].

Globally, BVDV-1b is the predominant subgenotype, followed by 1a, whereas BVDV-2 strains are most frequently of subgenotype 2a [7]. In contrast, a molecular survey compiling data from some Brazilian regions between 1998 and 2018 reported a predominance of BVDV-1 (54.4%), mainly subgenotype 1a, and BVDV-2b (25.7%) [24]. This divergence from the global pattern underscores the need for continuous molecular surveillance to detect recent shifts in BVDV subgenotype distribution.

The federative state of Rio Grande do Sul (RS) is one of the most traditional cattle-producing regions in Brazil, ranking seventh in herd size nationwide, with approximately 11.37 million head of cattle [25]. The state is also characterized by a predominance of *Bos taurus* breeds, in contrast to most other Brazilian regions where *Bos indicus* cattle are more common [26]. These features make RS a strategic area for genomic surveillance.

Given the substantial impact of BVDV on cattle health and productivity, the epidemiological importance of southern Brazil, and the absence of coordinated national control measures, this study aimed to conduct genomic surveillance of BVDV in Rio Grande do Sul State. The current distribution of viral subgenotypes was compared with data from a previous regional survey 10 years ago [27] to evaluate temporal changes in viral diversity, identify potential introductions of novel lineages, and provide updated insights to guide future control and prevention strategies in Brazil’s cattle industry. Furthermore, characterizing the circulating strains is essential for assessing the effectiveness of available vaccines and supporting the development of future control programs, reinforcing the need for continuous and comprehensive genomic surveillance [28].

## 2. Materials and Methods

### 2.1. Study Area

The study was conducted in the state of Rio Grande do Sul (RS), located in the extreme southern region of Brazil, which holds the seventh largest cattle population in the country, with approximately 11 million animals [25]. The state is an important center for both beef and dairy production, and its geographical location bordering Uruguay and Argentina highlight its relevance for regional animal health surveillance. The diversity of production systems, high herd density, and intense animal movement contribute to the epidemiological importance of RS for the circulation and persistence of BVDV.

### 2.2. Target Population and Sample Size

A total of 16,198 bovine serum samples analyzed in this study were obtained in 2020 through a national surveillance coordinated by the Department of Animal Health (DSA/SDA) of the Brazilian Ministry of Agriculture, Livestock and Food Supply (MAPA). This surveillance is designed to demonstrate the absence of foot-and-mouth disease virus (FMDV) circulation in the national cattle population, in compliance with the standards established by the WOAH for maintaining Brazil’s FMD-free status. Animals aged 6 to 24 months were randomly selected across different regions of Rio Grande do Sul [29]. Data regarding herd production type (beef or dairy) and vaccination status were not recorded. All serum samples were kept at −80 °C until laboratory processing.

### 2.3. RNA Isolation and RT-qPCR

The 16,198 bovine serum samples collected in Rio Grande do Sul were tested for BVDV RNA.

RNA isolation was performed using the MagMAX™ CORE Nucleic Acid Purification Kit (Applied Biosystems, Waltham, MA, USA) on the KingFisher™ 96 Flex Purification System (Thermo Fisher Scientific, Waltham, MA, USA), following the manufacturer’s recommendations. This semi-automated magnetic bead-based platform enables large-scale surveillance, allowing the simultaneous processing of up to 96 samples in approximately 30 min.

For RT-qPCR screening, pools of 100 were tested using the VetMAX™ BVDV 4ALL Kit (Thermo Fisher Scientific, Waltham, MA, USA), following the manufacturer’s recommendations. This assay allows broad detection of all known bovine pestivirus species, including BVDV-1, BVDV-2, and HoBiPeV. This pooling strategy was adopted to enable large-scale surveillance while maintaining cost-effectiveness [30]. The RT-qPCR assay used supports pooling of up to 100 samples, according to the manufacturer’s specifications. To evaluate the potential impact of pooling on diagnostic sensitivity, internal validation tests were performed using both field samples, which typically present lower viral loads, and experimentally diluted positive samples, including BVDV-1 (NADL), BVDV-2 (SV253), and HoBiPeV isolates propagated in cell culture. The assay was able to detect viral RNA in pools simulating dilutions up to 1:100. These results support the suitability of the pooling strategy for large-scale surveillance under the conditions of this study.

Positive pools were subsequently retested in pools of 10 with the VetMAX™ Gold PI Detection Kit (Thermo Fisher Scientific, Waltham, MA, USA). Individual positive samples from positive 10-sample pools were confirmed by conventional PCR. An internal positive control (IPC) was included during nucleic acid purification to ensure RNA extraction efficiency. Each run included two negative controls (serum from a BVDV-negative animal and a no-template control with ultrapure water) and two positive controls (BVDV-positive serum and the kit-provided control).

For confirmation and molecular characterization, individual samples from positive pools were subjected to conventional PCR targeting the 5′UTR and N^pro^ regions of the pestivirus genome. Complementary DNA (cDNA) synthesis was performed using the High-Capacity cDNA Reverse Transcription Kit (Applied Biosystems, Waltham, MA, USA). PCR amplification was conducted with Platinum™ Taq DNA Polymerase (Invitrogen, Carlsbad, CA, USA), following the manufacturer’s instructions. Primers set used for amplification are listed in Table 1.

### 2.4. Sanger Sequencing and Phylogenetic Analysis

The genetic variability of pestiviruses was assessed by sequencing fragments of the 5′UTR and N^pro^ genomic regions amplified from positive samples with the primer sets listed in Table 1. Amplicons were purified with the PureLink™ PCR Purification Kit (Thermo Fisher Scientific, Waltham, MA, USA), following the manufacturer’s instructions. Sanger sequencing was performed on an ABI PRISM^®^ 3100 Genetic Analyzer using the BigDye™ Terminator v3.1 Cycle Sequencing Kit (Thermo Fisher Scientific, Waltham, MA, USA). Partial sequences were assembled and edited in Geneious Prime v.2025.2.1 (Biomatters, Auckland, New Zealand). Nucleotide identity was determined using BLASTn (NCBI, Bethesda, MD, USA; https://blast.ncbi.nlm.nih.gov/Blast.cgi; accessed on 3 September 2025), web-based tool. Reference pestivirus sequences were retrieved from GenBank (https://www.ncbi.nlm.nih.gov/genbank/; accessed on 10 September 2025) for phylogenetic comparisons and were selected to represent the main subgenotypes relevant to the strains identified in this study, allowing clear phylogenetic interpretation while avoiding unnecessary tree complexity. Multiple sequence alignments were generated using Fast Fourier Transform (MAFFT v.7) [32] with default parameters. Phylogenetic trees were inferred using the maximum likelihood (ML) method implemented in the IQ-TREE web server (http://iqtree.cibiv.univie.ac.at) [33], under the best-fit substitution model automatically selected by ModelFinder [34]. The general time reversible model with gamma distribution and invariant sites (GTR + G + I) was applied when indicated [35,36]. Branch support was estimated with 1000 bootstrap replicates. The partial 5′UTR and N^pro^ sequences obtained in this study were deposited in GenBank under accession numbers PX503844-PX503892 (5′UTR) and PX503893-PX503916 (N^pro^).

### 2.5. Data Analysis

Data were expressed as absolute frequencies (*n*) and percentages (%). The distribution of BVDV subgenotypes (1a, 1b, 1d, and 2b) was compared between years (2010 and 2020) using the chi-square (χ^2^) test. When expected frequencies were low, Fisher’s exact test was applied as a complementary analysis.

Additionally, post hoc pairwise comparisons were performed to evaluate differences in the proportion of each subgenotype relative to the combined remaining subgenotypes between years. Bonferroni correction was applied to adjust for multiple comparisons.

Proportions and their 95% confidence intervals (CI) were calculated using the exact binomial method. Statistical significance was set at *p* < 0.05. All analyses were performed using Minitab Statistical Software, version 20 (Minitab LLC, State College, PA, USA).

## 3. Results

### 3.1. Detection of Pestivirus RNA in Cattle from Rio Grande do Sul

**Figure 1:**
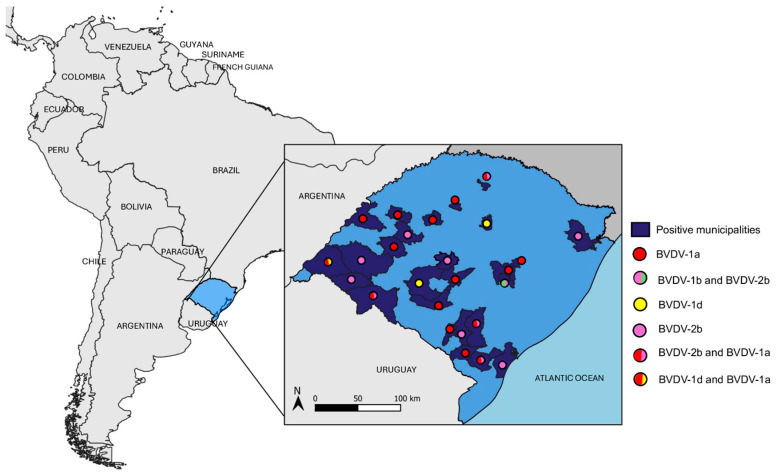
Geographic distribution of pestivirus-positive municipalities in Rio Grande do Sul, Brazil. Municipalities with at least one BVDV-positive sample are highlighted in dark blue. Colored dots indicate the pestivirus genotypes detected in each municipality.

A total of 16,198 bovine serum samples collected in Rio Grande do Sul in 2020 were screened for pestivirus RNA. Forty-nine samples (0.30%) tested positive by RT-qPCR and were confirmed by conventional RT-PCR. The municipalities harboring positive samples were mapped to visualize the spatial distribution of the detected pestivirus genotypes. Municipalities with at least one positive animal are highlighted in dark blue, while colored circles represent the genotypes identified in each location (Figure 1). Detailed information for all samples analyzed in this study (n = 16,198), including municipality of origin, animal sex, and age, is provided in Supplementary Table S1. The map was created using the free and open-source geographic information system QGIS (v.3.4) [37].

### 3.2. Molecular Characterization and Subgenotype Distribution

**Figure 2:**
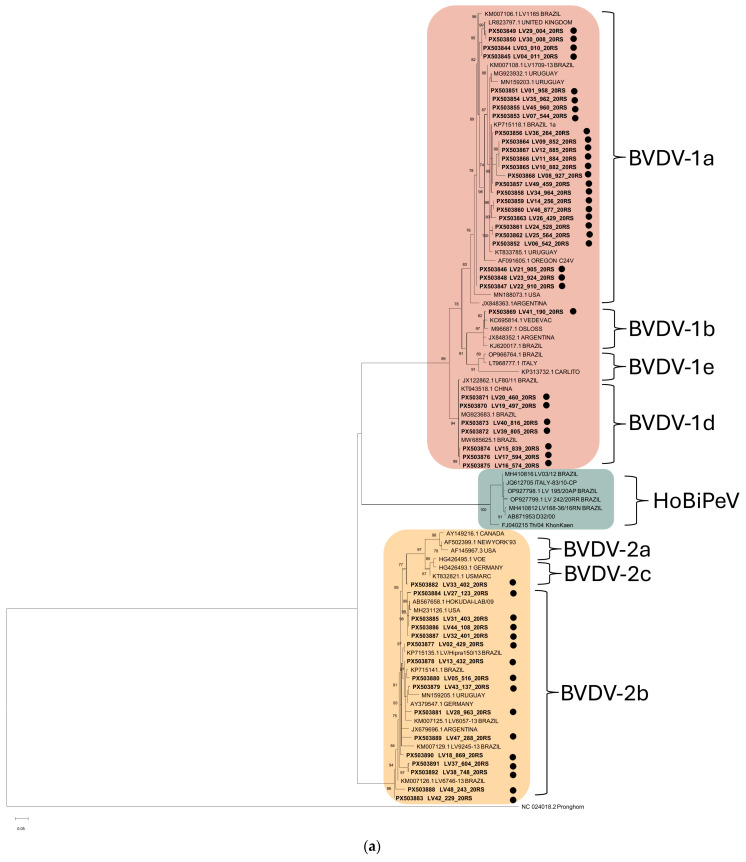

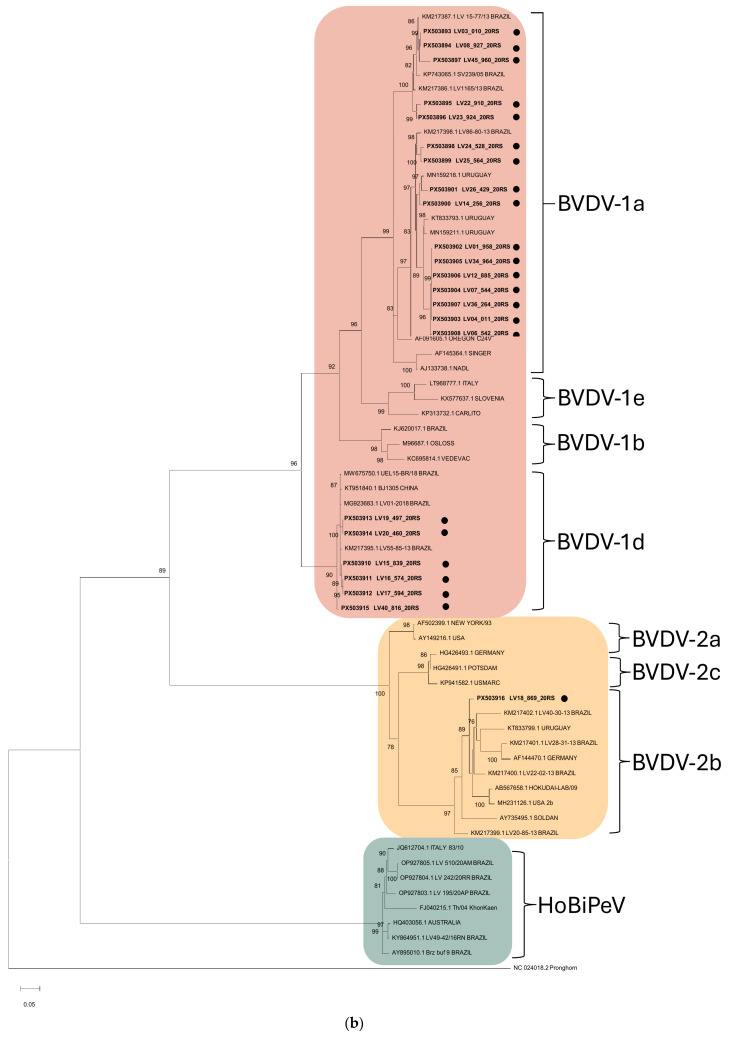
Maximum likelihood phylogenetic trees based on partial sequences of the 5′UTR (**a**) and N^pro^ (**b**) genomic regions of pestivirus detected in bovine serum samples from Rio Grande do Sul, southern Brazil. Trees were constructed from 244-nt (**a**) and 356-nt (**b**) multiple sequence alignments. Sequences generated in this study are indicated by circles (●). Bootstrap (n = 1000 replicates) values > 70% are shown at the nodes.

Partial sequencing of the 5′UTR and N^pro^ regions confirmed that all positive samples corresponded to BVDV-1 or BVDV-2 species. No HoBiPeV RNA was detected. The subgenotypes identified included BVDV-1a (25/49; 51%), BVDV-1b (1/49; 2%), BVDV-1d (7/49; 14%), and BVDV-2b (16/49; 33%). Phylogenetic analyses of the two genomic regions yielded consistent clustering with reference strains, providing robust bootstrap support for species and subgenotype level classification (Figure 2).

### 3.3. Temporal Comparison with Previous Data (2010)

**Figure 3:**
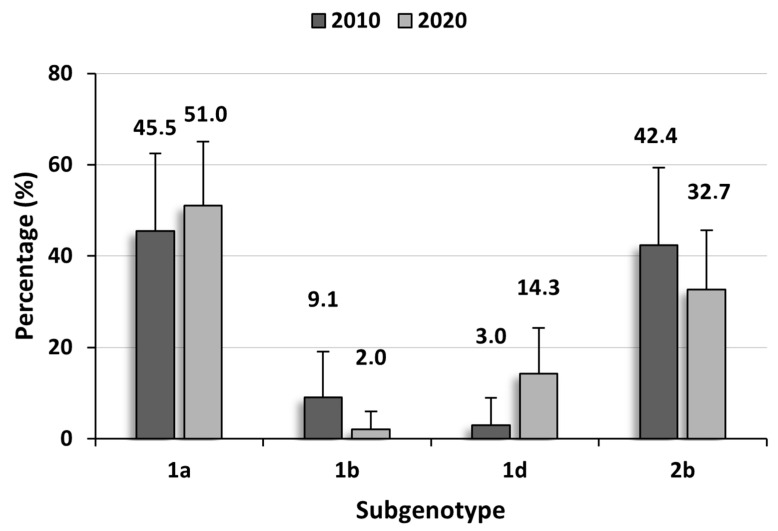
Relative frequency of BVDV subgenotypes detected in PCR-positive samples from Rio Grande do Sul in 2010 (n = 33) and 2020 (n = 49). Bars represent the proportion of each subgenotype identified in the two sampling periods. Error bars represent 95% confidence intervals. HoBiPeV was not detected in either dataset. Data for 2010 were obtained from Weber et al. 2014 [27].

When compared with data generated a decade earlier, the distribution of BVDV subgenotypes in Rio Grande do Sul [27] showed some variation over time. BVDV-1a remained the most frequently detected subgenotype in both periods, accounting for 45% (95% CI: 28–62) of positive samples in 2010 and 51% (95% CI: 37–65) in 2020. BVDV-1b was detected at low frequency in both periods, representing 9% (95% CI: 2–24) in 2010 and 2% (95% CI: 0–11) in 2020. The proportion of BVDV-1d was higher in the recent survey, increasing from 3% (95% CI: 0–16) in 2010 to 14% (95% CI: 4–27) in 2020. In contrast, BVDV-2b accounted for 42% (95% CI: 26–60) in 2010 and 33% (95% CI: 20–48) in 2020. No HoBiPeV was detected in either survey. Differences between years were not statistically significant (χ^2^ = 5.21, df = 3, *p* = 0.157; Figure 3).

## 4. Discussion

This study provides an updated molecular and epidemiological perspective on bovine pestivirus circulation in Rio Grande do Sul, a key cattle-producing region in southern Brazil. The findings show that BVDV-1a and BVDV-2b remain the most frequently detected subgenotypes in the region, while BVDV-1d was observed at a higher proportion than reported a decade ago [27]. The continued absence of HoBiPeV in both surveys suggests a consistent regional pattern of pestivirus circulation. In contrast, the lower proportion of BVDV-2b detections in the recent dataset represents a descriptive difference compared with earlier data.

These descriptive differences may be influenced by a combination of factors, such as local patterns of viral introduction, management practices, and regional variation in control measures across Brazil. For example, cattle production in Rio Grande do Sul is largely based on *Bos taurus* breeds [26], whereas in most other regions of the country *Bos indicus* cattle and water buffalo (*Bubalus bubalis*) predominate [38], which may influence patterns of viral circulation and introduction.

Although BVDV-1a is included in commercial vaccines used in Brazil, the predominance of this subgenotype in Rio Grande do Sul may relate to antigenic differences between vaccine strains and the field variants circulating in the region. Experimental evidence has shown that responses elicited by vaccines containing the reference strain NADL do not uniformly cross-neutralize genetically diverse BVDV-1a lineages [39], suggesting that immunity may be more effective against the vaccine strain than against heterologous variants present in regional herds. In addition, previous phylogenetic studies have demonstrated that BVDV-1 encompasses multiple clades with considerable genetic and antigenic divergence, including variation within the E2 region, a key determinant of neutralizing antibody responses [40]. This diversity within BVDV-1a may further contribute to the persistence of this subgenotype in the state, particularly in cattle populations with low vaccination coverage. Although the two datasets are not spatially matched and therefore not directly comparable in a statistical sense, the descriptive differences observed over the decade provide valuable insights into pestivirus circulation in Rio Grande do Sul. The relatively wide confidence intervals observed in some subgenotypes reflect the limited number of positive samples and should be considered when interpreting temporal differences. Overall, the overlapping 95% confidence intervals across subgenotypes, together with the absence of statistically significant differences, indicate that the observed variations between 2010 and 2020 should be interpreted as descriptive trends rather than true epidemiological changes.

A higher proportion of BVDV-1d detection was observed in the recent dataset, although this difference was not statistically supported. BVDV-2b remained one of the predominant subgenotypes, a distribution pattern that differs from what is commonly reported in other regions worldwide. The absence of HoBiPeV in both surveys is consistent with previous findings and suggests rare detection of this virus in Rio Grande do Sul, where only sporadic reports have been described, including its identification in water buffalo in the state [41]. These descriptive observations may reflect region-specific factors influencing viral circulation and highlight the importance of maintaining genomic surveillance to monitor changes over time.

The higher proportion of BVDV-1d detected in the recent dataset may be associated with sporadic introduction events, but its presence over time in Rio Grande do Sul could also relate to broader immunological or management factors in the region. Partial immunity induced by vaccines that include only a limited set of BVDV strains, commonly BVDV-1a (NADL), BVDV-1b (KE-9), and BVDV-2a (NY-93) in commercial formulations available in Brazil, may not provide full cross-protection against antigenically distinct subgenotypes, potentially creating selective pressures that allow these lineages to persist or expand within the population [42]. Continuous genomic monitoring will be important to clarify whether the proportion of BVDV-1d remains stable or changes as part of future patterns of pestivirus circulation.

Despite its low detection frequency in the present survey, BVDV-1b has recently been associated with outbreaks involving persistent infection, reproductive losses, and congenital malformations in beef cattle in neighboring Santa Catarina State [43], highlighting its potential epidemiological relevance in southern Brazil, particularly given the geographic proximity and frequent movement of cattle between these states.

Although the proportion of BVDV-2b detections was lower than that reported in 2010, this subgenotype remained comparatively frequent in the recent dataset and continues to represent a frequent component of the pestivirus population in the extreme southern region of Brazil [27]. The BVDV-2b sequences generated in this study grouped with previously reported Brazilian and Uruguayan isolates, showing phylogenetic proximity to strains circulating in neighboring areas of the southern cone. This pattern may be influenced by regional livestock movement and the long-standing connectivity between cattle-producing areas of extreme southern Brazil and adjacent countries.

The epidemiological relevance of BVDV-2b in southern Brazil is further supported by reports from neighboring Santa Catarina State, where this subgenotype has been detected in abortion cases and fetal lesions in cattle [44]. Together, these findings indicate that BVDV-2b circulates across different production systems in the southern Brazil and highlight the importance of integrated genomic surveillance across neighboring states to better understand the regional dynamics of pestivirus circulation.

While commercial vaccines generally include BVDV-2a, the BVDV-2b subgenotype predominates in extreme southern Brazil and exhibits antigenic differences relative to 2a, particularly in the E2 region [45]. Studies have reported variable cross-neutralization between these subgenotypes, which may influence the persistence of BVDV-2b in areas where vaccination is widespread [39]. Together, these observations highlight the importance of aligning vaccine antigen composition with the genetic diversity of locally circulating pestiviruses.

HoBiPeV was not detected in the present work, although the protocol described in this paper was able to detect them. For many years, Santa Catarina was the only Brazilian state officially recognized as free of foot-and-mouth disease (FMD) without vaccination, enforcing strict animal movement controls between states [46]. These restrictions may have limited pestivirus exchange from other Brazilian regions where HoBiPeV circulates, thereby helping preserve a distinct viral profile in Rio Grande do Sul. Additionally, the predominance of taurine breeds in RS herds, as opposed to the zebuine dominated systems characteristic of other parts of the country [26,38], may contribute to differences in animal contact patterns, management practices, and host–virus interactions, which could influence local viral ecology. Together, these contextual factors might help explain, but do not confirm, the enduring absence of HoBiPeV in the region.

The 5′UTR and N^pro^ phylogenies showed high concordance in subgenotype classification, confirming the reliability of these genomic targets for surveillance purposes. The slightly lower bootstrap support observed in the 5′UTR tree compared to N^pro^ is consistent with previous reports [17]. Therefore, despite its lower discriminatory power at the subgenotype level, the 5′UTR remains a robust and practical marker for large scale screening and initial molecular characterization of pestivirus populations. Amplification success varied among genomic regions, with N^pro^ amplified in 24 out of 49 samples. This reduced performance likely reflects primer target mismatches and natural sequence variation within the region, which can hinder efficient amplification, particularly given that N^pro^ is more variable and less conserved than the 5′UTR. Similar challenges have been reported in other pestivirus studies [47]. Although RNA integrity was confirmed using an internal control (GAPDH), a substantial proportion of samples could not be amplified despite repeated attempts, especially in samples with low viral loads commonly observed under field conditions. Therefore, the use of the 5′UTR region allowed broader sample coverage, while N^pro^ sequencing was limited to a subset of samples suitable for higher-resolution phylogenetic analysis.

The implications of these findings extend beyond molecular epidemiology. Brazil’s success in eradicating FMD demonstrates its capacity to implement coordinated, science-based animal health programs [46]. Applying similar measures to pestivirus control could build upon existing infrastructure for surveillance, laboratory diagnostics, and animal movement regulation. In addition, wildlife may act as a potential reservoir in extensive livestock systems, as BVDV RNA has been detected in free-ranging wild boars in Brazil [48], highlighting the need for integrated surveillance across domestic and wild hosts.

Importantly, most BVDV vaccines licensed in Brazil are inactivated and contain only BVDV-1a, BVDV-1b and/or BVDV-2a [39]. The continued use of vaccines and diagnostic assays formulated for these strains may not adequately reflect the viral diversity present in Brazilian herds. As a result, infections caused by antigenically distinct subgenotypes may be underdiagnosed, particularly when test performance is reduced for divergent strains [49]. The detection of BVDV-1d and 2b, subgenotypes absent from most commercial vaccines, raises concerns about incomplete immune coverage. Under immunological pressure induced by vaccines targeting a set of variants, antigenically distinct lineages may gain a selective advantage, expanding within vaccinated populations [50]. Incorporating genomic surveillance into vaccine strain selection and efficacy assessment will be crucial to close these immunological gaps and ensure durable protection against the evolving pestivirus landscape.

Recent experimental work has shown that antigenic heterogeneity among bovine pestiviruses can substantially influence serological responses to vaccination, highlighting that cross-neutralization against genetically divergent subgenotypes may be limited [38]. In this context, the continued high proportion of BVDV-2b observed in Rio Grande do Sul may be influenced, at least in part, by the antigenic composition of the commercial vaccines commonly used in Brazil. These findings provide a conceptual framework for interpreting how immunological pressures could contribute to the persistence of certain subgenotypes within the regional viral population.

Altogether, these findings reinforce the importance of continuous molecular monitoring of pestiviruses in Brazil. The low proportion of RT-qPCR-positive samples observed in this study is consistent with previous reports from different regions of Brazil, which have also described low frequencies of active pestivirus infection despite evidence of widespread viral circulation [27,51,52]. Similar patterns have been reported in other endemic settings worldwide, where molecular detection rates are typically low in cross-sectional studies [22].

In contrast, serological investigations in Brazil frequently demonstrate high levels of exposure, indicating that pestiviruses are widely distributed in cattle populations [24,51]. This apparent discrepancy between low molecular detection and high seroprevalence suggests that most animals are not actively infected at the time of sampling but have been previously exposed or immunized.

Therefore, the low RT-qPCR positivity observed in the present study likely reflects the epidemiological dynamics of pestivirus infection in endemic settings, rather than a true absence of viral circulation. This pattern is consistent with a scenario of stable endemicity, in which viral transmission persists at low detectable levels while a large proportion of the population has pre-existing immunity.

However, some methodological aspects should be considered when interpreting these findings. The use of large pooling strategies may reduce analytical sensitivity, and false-negative results cannot be completely excluded, particularly in samples with low viral loads [22]. In addition, the samples analyzed in this study were derived from a FMD surveillance program not specifically designed for pestivirus investigation. Therefore, the sampling approach may introduce some potential sources of selection bias, particularly due to the defined age range and the limited availability of epidemiological data.

Despite these limitations, the genetic composition of the viral population appears dynamic, with evidence of lineage persistence, gradual replacement, and the emergence of previously minor subgenotypes. Sustained genomic surveillance, coupled with updated vaccination strategies that reflect circulating variants, is essential to mitigate BVDV spread and its associated economic losses.

## 5. Conclusions

This study provides an updated overview of BVDV genetic diversity in Rio Grande do Sul. BVDV-1a remained the most frequently detected subgenotype, and BVDV-2b represented a substantial proportion of the sequences, a pattern that differs from the global predominance of BVDV-2a. A higher proportion of BVDV-1d was observed in the recent dataset compared with the survey conducted a decade earlier; however, this difference was not statistically supported and should be interpreted with caution. No HoBiPeV was detected, consistent with previous findings for this region [27], in contrast to other Brazilian regions where HoBiPeV has been more commonly detected in cattle populations [51,52]. Overall, the results suggest a relatively stable regional BVDV profile over the past decade, with only minor descriptive variations in subgenotype frequencies. These findings highlight the importance of sustained genomic surveillance to monitor potential changes in viral circulation and to support future control strategies in Brazilian cattle herds.

## Figures and Tables

**Table 1:** Primers used for conventional PCR targeting the 5′UTR and N^pro^ regions of pestiviruses.

Primer Pair	Target Region	Target Size (bp)	Sequence (5′–3′)	Reference
324–326	5′UTR	288	F: ATGCCCWTAGTAGGACTAGCA R: TCAACTCCATGTGCCATGTAC	[31]
BD1–BD3	N^pro^	428	F: TCTCTGCTGTACATGGCACATG R: CCATCTATRCACACATAAATGTGGT	[17]

## Data Availability

The nucleotide sequences generated in this study have been deposited in GenBank under accession numbers PX503844-PX503892 (5′UTR) and PX503893-PX503916 (N^pro^).

## Supplementary Materials

The following supporting information can be downloaded at: https://www.mdpi.com/article/10.3390/v18050498/s1, Table S1: Sampling information for all bovine serum samples analyzed in this study.
